# Supplementary material for: Poloxamer 407 Based Gel Formulations for Transungual Delivery of Hydrophobic Drugs: Selection and Optimization of Potential Additives
Source: Polymers (Basel). 2021 Sep 30;13(19):3376. doi: 10.3390/polym13193376 (PMC8512385; doi:10.3390/polym13193376)
Supplement: Supplementary file 1 [file polymers-13-03376-s001.zip › polymers-1386767-Supplementary.pdf]

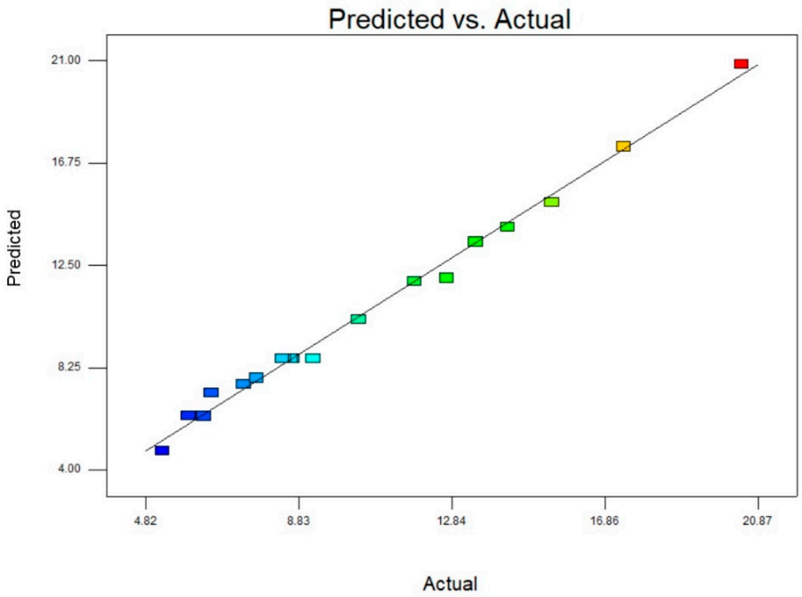

Supplementary figure S1. Design experts plot between predicted and actual values for gelation temperature

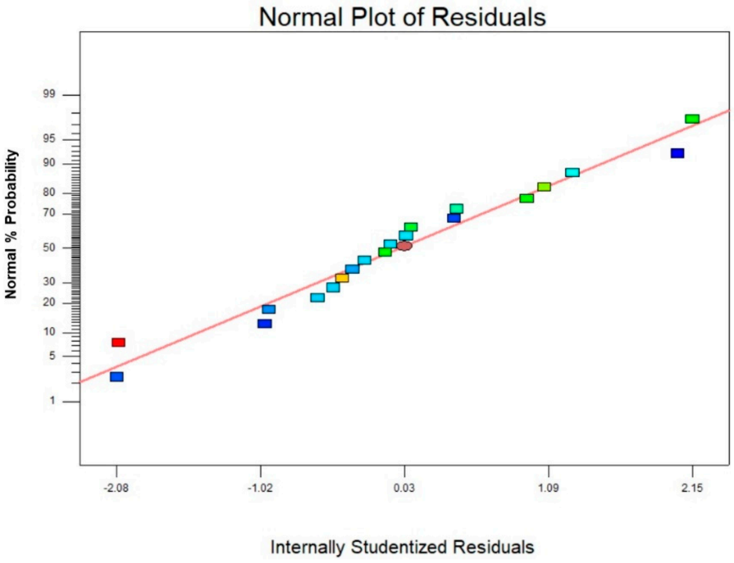

Supplementary figure S1. Normal plot of residuals for gelation temperature

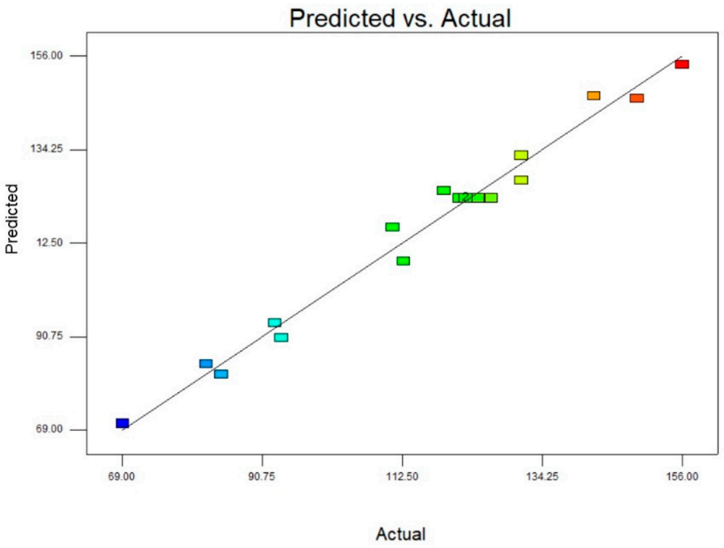

Supplementary figure S3. Design experts plot between predicted and actual values for gel strength

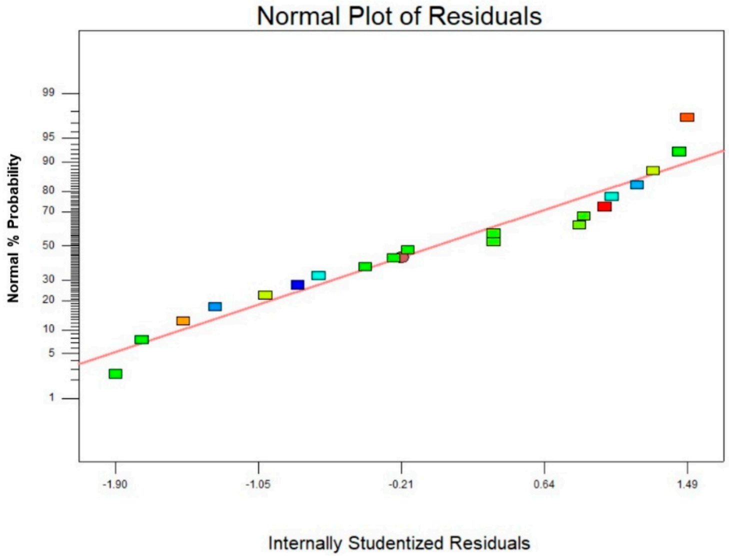

Supplementary figure S2. Normal plot of residuals for gel strength
